# Expression of PD-L1 in breast invasive lobular carcinoma

**DOI:** 10.1371/journal.pone.0309170

**Published:** 2024-10-10

**Authors:** Eunah Shin, Hye Min Kim, Ja Seung Koo

**Affiliations:** Department of Pathology, Yonsei University College of Medicine, Seoul, South Korea; The University of Queensland Faculty of Medicine, AUSTRALIA

## Abstract

**Purpose:**

The purpose of this study was to investigate the expression of PD-L1 in invasive lobular carcinoma (ILC) and to determine its implications.

**Methods:**

Tissue microarrays were constructed for 101 cases of ILC, and immunohistochemical staining for PD-L1 (using 22C3, SP142, and SP263 antibodies) was performed to examine the correlation between staining results and clinicopathologic parameters.

**Results:**

The positive cut-off values were defined as tumor cell (TC)≥1%, immune cell (IC)>0%, and IC≥1%. The range of PD-L1 TC positivity was 0.0–2.0%, with PD-L1 SP263 TC showing the highest positivity of 2.0%. The range of PD-L1 IC positivity was 0–21.8% for IC ≥ 1%, with PD-L1 22C3 IC showing the highest positivity. When PD-L1 IC was positive (IC≥1%), the highest antibody agreement was observed between SP263 and SP142 (OA = 93.1%), while the lowest agreement was observed between 22C3 and SP263 (OA = 73.3%, κ = 0.040). PD-L1 22C3 IC positivity (≥1%) was associated with high nuclear grade (p = 0.002), HER-2 positivity (p = 0.019), and pleomorphic type (p = 0.002).

**Conclusion:**

PD-L1 expression in ILC shows a low TC positivity rate (0–2%) with various antibody clones and a variable IC positivity rate (0–21.8%). Pleomorphic type ILC exhibits higher PD-L1 IC positivity.

## Introduction

Breast cancer is the most common cancer among women, and invasive carcinoma is classified into various histological subtypes, but first it is largely divided into invasive ductal carcinoma (IDC) and invasive lobular carcinoma (ILC) [1]. Among these, ILC accounts for about 5–15% of invasive carcinomas [2,3], and in recent years, its incidence has been increasing compared to IDC due to the increase in hormone replacement therapy and alcohol consumption [4,5]. Pleomorphic type of ILC is an aggressive subtype of ILC [6,7], and it exhibits higher expression of p53 and human epidermal growth factor receptor (HER-2), while showing lower expression of hormone receptors [8]. It is also reported to occur in older women. ILC differs from IDC in several aspects, clinically showing multiplicity and bilaterality [9,10], and histologically exhibiting non-cohesive cancer cells due to the loss of E-cadherin expression [11]. Also, the metastasis pattern of ILC is different from IDC, mainly involving bone, gastrointestinal tract, uterus, meninges, and ovary, and showing diffuse serosal involvement [10,12,13]. In addition to the difference in metastatic sites, the ILC also tends to show more late recurrences compared to IDC. The increased rate of incidence of ILC can be attributed in part to recent advances in imaging and surveillance modalities such as magnetic resonance imaging (MRI) [14], positron emission tomography/computed tomography (PET/CT) [15], and digital mammography/tomosynthesis [16]. While the 5-year survival rate for ILC is more favorable when compared to IDC, survival rates beyond 5 years are reported to be worse [17], which can be most rightly explained by disseminated cancer cells remaining in a dormant state for an extended period of time [18]. Therefore, targeting the dormant metastatic niche can be proposed as an effective target for improving survival rates in ILC.

Programmed death 1 (PD-1) is a checkpoint molecule in immune reactions that is expressed in various immune cells [19]. PD-L1 is the ligand for PD-1, and it is expressed on tumor cells. Through the binding of PD-L1 and PD-1, tumor cells evade the antitumor immune response [20,21]. PD-L1 has been reported to be expressed in 20–70% of lung cancer [20,22–25], urinary bladder cancer [26], malignant melanoma [27], ovarian cancer [28], and other tumors. Therefore, targeted therapy for PD-L1 can induce antitumor immune response in tumors that express PD-L1. PD-L1 targeted therapy has been tested in preclinical and clinical trials for various cancer types [22–24,26,29–31], and anti-PD-L1 antibodies such as BMS-936559 [32] and MPDL3280A [23,26] have already been developed. As such, evaluation of PD-L1 expression is important for targeted therapy, and the most common and convenient method for investigating PD-L1 expression is immunohistochemistry (IHC) using monoclonal PD-L1 antibodies in formalin-fixed paraffin-embedded (FFPE) tissue. Commercially available monoclonal PD-L1 antibodies include clones 28–8 [33], 22C3 [34], SP142 [23,26], E1L3N [35,36], and others. Previous studies have reported lower levels of tumor-infiltrating lymphocytes (TILs) in ILC compared to IDC [37]. However, it has been noted that a significant proportion of ILC cases exhibit infiltration of immune cells, with 49.0% showing stromal TILs at 5–10% and 23.5% with TILs exceeding 10% [37]. Additionally, expression of PD-L1 in TILs of breast cancer has been documented, bearing various clinical implications, and thereby highlighting the need for research on PD-L1 expression in ILC [38–40]. While there have been many studies on PD-L1 expression in breast cancer, research on PD-L1 expression in ILC has been limited. Thus, the aim of this study was to evaluate PD-L1 expression in ILC and to investigate its implication.

## Material and methods

### Patient selection and clinicopathologic evaluation

We utilized FFPE tissue samples from patients diagnosed with ILC who underwent surgery at Severance Hospital from January 2000 to December 2012. The study adhered to the principles of the Declaration of Helsinki and obtained approval from the Institutional Review Board of Yonsei University Severance Hospital (IRB number:4-2023-1321). Due to the retrospective nature of the study, patient consent was exempted by the Institutional Review Board of Yonsei University Severance Hospital. We accessed the medical records or archived samples for research purpose until 31 December 2023. Authors did not have access to the information that could identify individual participants during or after data collection. Cases that received chemotherapy prior to surgery were excluded. All cases were retrospectively reviewed by a breast pathologist (Koo JS), and H&E-stained slides were reviewed for histology. Classic type ILC is defined by small, round, regular tumor cells diffusely infiltrating and forming single filed lines between collagen bundles. Pleomorphic ILC, while exhibiting the same infiltrating pattern as classic type ILC, shows more pleomorphic tumor cell nuclei in more than 50% of the tumor. These nuclei display greater contour irregularity, more prominent nucleoli, increased chromatin, and/or greater nuclear size (up to four times larger than lymphocytes [6]. The histological grade was assessed using the Nottingham grading system [41]. TILs were assessed following the guidelines of the international TILs working group [42]. TILs were categorized into three intervals: less than 5%, 5–10%, and greater than 10% [37]. Disease-free survival (DFS) was calculated from the date of the first curative surgery to the date of the first loco-regional or systemic relapse, or death without any type of relapse. Overall survival (OS) was estimated from the date of the first curative operation to the date of the last follow-up or death from any cause. Clinicopathologic parameters evaluated in each breast cancer included patient age at initial diagnosis, lymph node metastasis, tumor recurrence, distant metastasis, and patient’s survival.

### Tissue microarray

After reviewing the H&E-stained slides, the most appropriate paraffin block of the tumor was retrieved for each case. The most representative tumor area was then marked on the paraffin block, and the punch machine was used to extract two 3mm sized cores from the selected area of each case. Then the cores were inserted into a 6x5 recipient block.

### Immunohistochemistry

The IHC antibodies used in this study are listed in S1 Table. FFPE tissue sections were used for IHC. Tissue sections with a thickness of 3μm were made from paraffin blocks, deparaffinized and rehydrated using xylene and alcohol solutions, and subjected to antigen retrieval using CC1 buffer (Cell Conditioning 1; citrate buffer Ph 6.0, Ventana Medical System). IHC was performed using the Ventana Discovery XT automated stainer (Ventana Medical System, Tucson, AZ, USA) and included appropriate positive and negative controls.

### Interpretation of immunohistochemical results

A cut-off value of 1% or more positively stained nuclei was used to define ER and PR positivity [43]. HER-2 staining was analyzed according to the American Society of Clinical Oncology (ASCO)/College of American Pathologists (CAP) guidelines using the following categories: 0 = no immunostaining; 1+ = weak incomplete membranous staining, less than 10% of tumor cells; 2+ = complete membranous staining, either uniform or weak in at least 10% of tumor cells; and 3+ = uniform intense membranous staining in at least 10% of tumor cells [44]. HER-2 immunostaining was considered positive when strong (3^+^) membranous staining was observed whereas cases with 0 to 1^+^ were regarded as negative. ILC cases showing 2+ HER-2 expression were evaluated for HER-2 amplification by FISH (fluorescence in situ hybridization).

In interpreting the results of PD-L1 IHC, the scoring method and cut-off value typically vary depending on the tumor type and antibody clones used [45,46]. However, in this study, we aimed to investigate differences attributable to antibody types by employing the same scoring system and cut-off value across different antibodies. Therefore, we utilized a scoring system based on tumor cells (TC) and immune cells (IC). PD-L1 expression was measured in TC and IC. TC was defined as the proportion (%) of tumor cells showing membranous staining of any intensity for PD-L1, while IC was defined as the proportion (%) of tumor area occupied by PD-L1 staining immune cells (lymphocyte, histiocyte, dendritic cell, and granulocytes). To compare the results of different antibodies, positive criteria were calculated for each clone as TC≥1% [47,48] and IC≥1% [49], respectively. The concordance of each antibody was compared by these criteria. In this study, three pathologists conducted interpretations of PD-L1 stained slides. Those cases in which the three pathologists did not confer, the final consensus was made by reviewing the slides under a multi-view microscope.

### Tumor phenotype classification

We classified breast cancer phenotypes according to the IHC results for ER, PR, HER-2, and Ki-67 labelling index. FISH results for HER-2 were as follows [50]: *luminal A type*: ER and/or PR positive, HER-2 negative, and Ki-67 labelling index <14%; *luminal B type*: (HER-2 negative) ER and/or PR positive, HER-2 negative, and Ki-67 labelling index ≥14% and (HER-2 positive) ER and/or PR positive and HER-2 overexpressed and/or amplified; *HER-2 type*: ER and PR negative and HER-2 overexpressed and/or amplified; *TNBC type*: ER, PR, and HER-2 negative.

### Statistical analysis

Data were statistically processed using SPSS for Windows version 12.0 (SPSS Inc., Chicago, IL). Student’s *t* test and Fisher’s exact test were used for continuous and categorical variables, respectively. Statistical significance was assumed when *P*<0.05. Cohen’s kappa coefficient was used to assess the agreement between any two PD-L1 antibody clones for each scoring method and was interpreted as: <0, no agreement; 0.0–0.20, slight agreement; 0.21–0.40, fair agreement; 0.41–0.60, moderate agreement; 0.61–0.80, substantial agreement; 0.81–1.00, almost perfect agreement [51].

## Results

### Characteristics of ILC

S2 Table shows the clinicopathologic characteristics of 101 cases of ILC, including 91 cases of classic type and 10 cases of pleomorphic type. The pleomorphic type was associated with older age (p = 0.033), higher nuclear grade (p<0.001), higher histologic grade (p<0.001), higher T stage (p = 0.026), higher TIL (≥5%, p = 0.020), PR negativity (p = 0.005), HER-2 positivity (p = 0.002), higher Ki-67 labelling index (p<0.001), and non-luminal A subtype (p<0.001) compared to the classic type.

### Expression of PD-L1 in ILC

**Fig 1:**
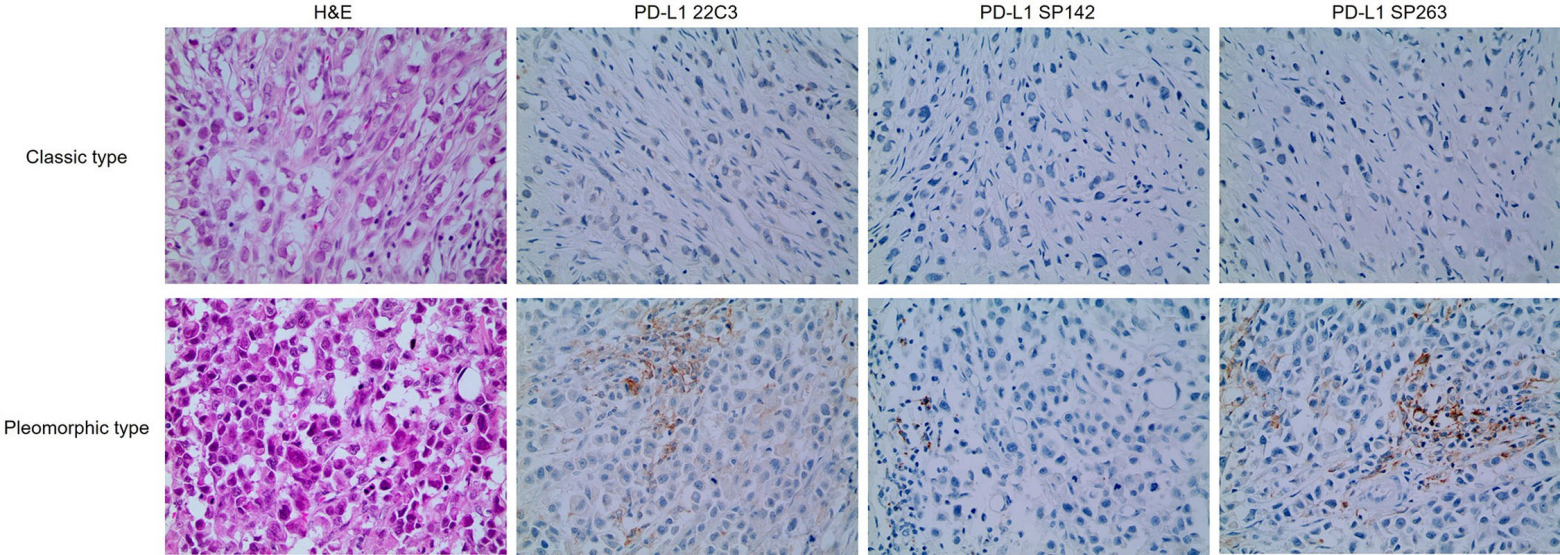
Expression of PD-L1 in ILC. The expression of PD-L1 in immune cells is higher in pleomorphic type than in classic type when stained with PD-L1 SP263.

According to the investigation of PD-L1 expression using 22C3, SP142, and SP263 antibodies in ILC, staining was almost absent in tumor cells of ILC with a range of 0–2%. For IC, depending on the antibody clone, IC≥1% was observed in the range of 0–21.8% (Table 1). There were significant differences in PD-L1 SP263 IC (p = 0.001) depending on the histologic type of ILC, with a higher proportion of PD-L1 IC positivity in the pleomorphic type (Table 1 and Fig 1).

**Table 1:** Expression of PD-L1 in ILC according to the cytologic type.

Parameters	Total N = 101 (%)	Classic type n = 91 (%)	Pleomorphic type n = 10 (%)	p-value
PD-L1 22C3 TC				n/a
<1%	101 (100.0)	91 (100.0)	10 (100.0)	
≥1%	0 (0.0)	0 (0.0)	0 (0.0)	
PD-L1 22C3 IC				0.342
<1%	79 (78.2)	70 (76.9)	9 (90.0)	
≥1%	22 (21.8)	21 (23.1)	1 (10.0)	
PD-L1 SP142 TC				n/a
<1%	101 (100.0)	91 (100.0)	10 (100.0)	
≥1%	0 (0.0)	0 (0.0)	0 (0.0)	
PD-L1 SP142 IC				n/a
<1%	101 (100.0)	91 (100.0)	10 (100.0)	
≥1%	0 (0.0)	0 (0.0)	0 (0.0)	
PD-L1 SP263 TC				0.189
<1%	99 (98.0)	90 (98.9)	9 (90.0)	
≥1%	2 (2.0)	1 (1.1)	1 (10.0)	
PD-L1 SP263 IC				*0*.*002*
<1%	94 (93.1)	87 (95.6)	7 (70.0)	
≥1%	7 (6.9)	4 (4.4)	3 (30.0)	

### Difference and concordance of PD-L1 expression in ILC according to PD-L1 antibody clones and scoring systems

When analyzing the differences in PD-L1 expression based on PD-L1 clones and scoring systems in ILC, PD-L1 expression was investigated in ILC using a PD-L1 positive cut-off value of TC≥1% and IC≥1%. The range of PD-L1 TC positivity was 0–2.0%, with PD-L1 SP263 TC showing the highest positivity rate of 2.0%. The range of PD-L1 IC positivity was 0–21.8% when IC was ≥1%, with PD-L1 22C3 IC showing the highest positivity rate (Table 2).

**Table 2:** PD-L1 positivity for TC and IC in ILC according to PD-L1 clones.

PD-L1 clone	TC≥1%, n (%)	IC≥1%, n (%)
22C3	0 (0.0)	22 (21.8)
SP142	0 (0.0)	0 (0.0)
SP263	2 (2.0)	7 (6.9)

When analyzing the concordance of PD-L1 expression between clones according to the scoring system (Table 3), there were only 2 cases showing PD-L1 expression with 22C3 when the positive cut-off was set at TC≥1%, which was not significant for the analysis. For IC≥1%, SP142 were negative in all cases, and the clones that showed concordance were 22C3 and SP263 with slight agreement (OA = 73.3%, κ = 0.040).

**Table 3:** Pairwise comparisons for concordance and kappa statistics among PD-L1 clones according to the scoring system.

Scoring system and PD-L1 clone pair	Overall agreement (OA) (%)	Kappa coefficient (95%CI)	Category of agreement
TC≥1%			
22C3 vs. SP142	101 (100.0)	n/a	n/a
22C3 vs. SP263	99 (98.0)	n/a	n/a
SP142 vs. SP263	99 (98.0)	n/a	n/a
IC≥1%			
22C3 vs. SP142	79 (78.2)	n/a	n/a
22C3 vs. SP263	74 (73.3)	0.040 (0.071)	Slight
SP142 vs. SP263	94 (93.1)	n/a	n/a

<0, no agreement; 0.0–0.20, slight agreement; 0.21–0.40, fair agreement; 0.41–0.60, moderate agreement; 0.61–0.80, substantial agreement; 0.81–1.00, almost perfect agreement.

### Correlation between PD-L1 status and clinicopathologic factors in ILC

**Fig 2:**
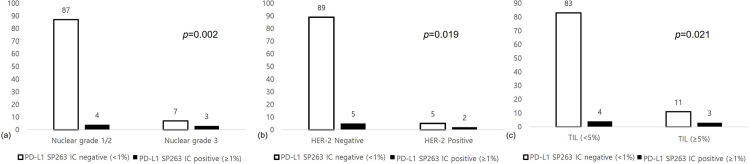
Correlation between PD-L1 status and clinicopathologic factors in ILC. When PD-L1 22C3 is IC positive (≥1%), it shows a higher proportion of high nuclear grade (a, p = 0.002), HER-2 positivity (b, p = 0.019), and higher TIL (≥5%) (c, p = 0.021) compared to negative cases.

The association between PD-L1 expression and clinicopathologic factors in ILC was investigated, and the results showed that PD-L1 SP263 IC status was associated with nuclear grade (p = 0.002), HER-2 status (p = 0.019), and TIL status (p = 0.021). Cases in which PD-L1 22C3 IC was positive (≥1%) showed a higher proportion of high nuclear grade, HER-2 positivity, and higher TIL (Fig 2).

### Impact of PD-L1 status on tumor recurrence and patient death in ILC

To investigate the impact of PD-L1 status on the prognosis of ILC, it is ideal to conduct survival. However, in this study, the PD-L1 positivity rate was very low, especially the PD-L1 TC positivity rate. Consequently, when descriptive statistics were applied instead of survival analysis, no statistically significant association was found between PD-L1 status and tumor recurrence or patient death (S3 Table).

## Discussion

In this study, the expression of PD-L1 was investigated using three antibodies (22C3, SP142, and SP263) in primary ILC. Previous studies on PD-L1 expression in ILC tumor cells have been limited, but one study reported a PD-L1 positivity rate of 17% in ILC tumor cells using B7-H1 and SP142 antibodies, with a positive cut-off value of more than 5% [52]. In previous studies on breast cancer in general, the PD-L1 TC positivity rate ranged from 21.7% to 56.6%, indicating that the PD-L1 TC positivity rate is lower in ILC compared to breast cancer as a whole [53]. In a previous study on ILC, all cases were reported to have PD-L1-positive TIL, with focal (<5%) positivity in 11% of cases, moderate (10–24%) positivity in 60% of cases, and diffuse (50–100%) positivity in 29% of cases, showing a higher PD-L1 IC positivity rate than in this study [52]. The PD-L1 IC positivity rate in breast cancer in general is usually reported to be 10–30% [54].

The expression of PD-L1 in tumors is influenced not only by analytic factors such as antibody clones, interpretation scoring systems, and cut-off values [55], but also by tumor intrinsic factors such as tumor stage, molecular subtype, and tumor stroma type [54]. Moreover, previous studies on the expression of SP142/SP263/22C3 in breast cancer have shown different results depending on the tumor origin (primary or metastatic) and tissue type (resection or TMA) used in the studies [56–59]. ILC, as a distinct histologic subtype of breast cancer, may show a different PD-L1 status from other breast cancer subtypes.

In this study, PD-L1 IC positivity was associated with poor prognostic factors such as high nuclear grade, HER-2 positivity, and pleomorphic type. However, in a previous study on ILC, tumor cell PD-L1 expression was associated with younger age but did not show any association with prognostic factors such as ER, PR, HER-2, grade, size, stage, and death [52]. In other tumors, PD-L1 expression on immune cells has been associated with a favorable prognosis [60]; in colorectal cancer, similar to ILC in our study, the presence of PD-L1 expression on immune cells has been linked to poor differentiation, lymphatic invasion, vascular invasion, and high budding grade [61]. It has been suggested that PD-L1-positive immune cells located at the invasive front may contribute to immune escape at the invasive edge, implying the possibility of similar associations in ILC. In a meta-analysis study on primary breast cancer, PD-L1 TC positivity was reported to be associated with ductal carcinomas, large tumor size, histological grade 3 tumors, high Ki-67 labelling index, triple-negative breast cancer, high TIL, and shorter DFS and OS. On the other hand, PD-L1 IC positivity was reported to be associated with better DFS and OS [62]. One of the limitations of this study is the relatively low proportion of ILC cases compared to IDC in breast cancer, resulting in limitations in the number of ILC cases included. Additionally, the number of cases showing PD-L1 TC positivity is limited, potentially leading to limitations in survival analysis and subgroup analysis. Therefore, it is necessary to conduct further studies to investigate the role of PD-L1 as a prognostic factor in the ILC subgroup, as the association between PD-L1 status and poor prognostic factors varies depending on the breast cancer subtype.

The most important significance of PD-L1 status in breast cancer is its predictive role for immune therapy. Currently, immune therapy such as pembrolizumab is approved for TNBC when PD-L1 22C3 IHC is CPS 10 or higher. In this study, PD-L1 22C3 positivity rates showed the highest values when IC was ≥1% (21.8%), indicating the need for clinical studies on the response to immune therapy based on PD-L1 22C3 IHC results in ILC. By analyzing genomic, transcriptomic, and proteomic data, ILC can be classified into immune-related subtype (IRS) and hormone-related subtype (HRS) [63]. ILS is characterized by mRNA up-regulation of PD-L1, PD-1, and CTLA-4, and immune cell infiltration. The proportion of IRS in ILC was found to be about 62%. Therefore, clinical studies are needed to investigate the possibility of using immune checkpoint inhibitors in IRS. Furthermore, since IRS is particularly sensitive to DNA-damaging agents in cell line studies, additional studies are needed to determine whether combination therapy of chemotherapy and immune therapy can provide further benefits, especially in IRS.

In conclusion, PD-L1 expression in ILC shows a low TC positivity rate (0–2%) across various antibody clones and a variable IC positivity rate (0–21.8%), and pleomorphic type ILC exhibits higher PD-L1 IC positivity.

## Supporting information

S1 Table Clone, dilution, and source of antibodies used. (DOCX)

S2 Table Clinicopathologic characteristics of invasive lobular carcinoma. (DOCX)

S3 Table Impact of PD-L1 status on tumor recurrence and patient death in ILC. (DOCX)
