# Supplementary material for: Expression of PD-L1 in breast invasive lobular carcinoma
Source: PLoS One. 2024 Oct 10;19(10):e0309170. doi: 10.1371/journal.pone.0309170 (PMC11466385; doi:10.1371/journal.pone.0309170)
Supplement: S1 Table — (DOCX) [file pone.0309170.s001.docx]

**Supplementary Table 1.** Clone, dilution, and source of antibodies used

| Antibody | **Company** | **Clone** | **Dilution** |
| --- | --- | --- | --- |
| *PD-L1 related* | | | |
| PD-L1 22C3 | DAKO, Glostrup, Denmark | 22C3 | N/A |
| PD-L1 SP142 | Ventana Medical Systems | SP142 | N/A |
| PD-L1 SP 263 | Ventana Medical Systems | SP263 | N/A |
| *Molecular subtype related proteins* | |  |  |
| ER | Thermo Scientific, San Siego, CA, USA | SP1 | 1:100 |
| PR | DAKO, Glostrup, Denmark | PgR | 1:50 |
| HER-2 | DAKO, Glostrup, Denmark | Polyclonal | 1:1500 |
| Ki-67 | Abcam, Cambridge, UK | MIB | 1:1000 |
