# Supplementary material for: Expression of PD-L1 in breast invasive lobular carcinoma
Source: PLoS One. 2024 Oct 10;19(10):e0309170. doi: 10.1371/journal.pone.0309170 (PMC11466385; doi:10.1371/journal.pone.0309170)
Supplement: S2 Table — (DOCX) [file pone.0309170.s002.docx]

| **Supplementary Table 2.** Clinicopathologic characteristics of invasive lobular carcinoma | | | | |
| --- | --- | --- | --- | --- |
| *Parameters* | Total  N=101 (%) | Classic type  n=91 (%) | Pleomorphic type  n=10 (%) | P-value |
| Age (years) |  |  |  | **0.014** |
| <50 | 57 (56.4) | 55 (60.4) | 2 (20.0) |  |
| ≥50 | 44 (43.6) | 36 (39.6) | 8 (80.0) |  |
| Nuclear grade |  |  |  | **<0.001** |
| 1/2 | 91 (90.1) | 91 (100.0) | 0 (0.0) |  |
| 3 | 10 (9.9) | 0 (0.0) | 10 (100.0) |  |
| Histologic grade |  |  |  | **<0.001** |
| I/II | 98 (97.0) | 91 (100.0) | 7 (70.0) |  |
| III | 3 (3.0) | 0 (0.0) | 3 (30.0) |  |
| T stage |  |  |  | **0.046** |
| T1 | 60 (59.4) | 57 (62.6) | 3 (30.0) |  |
| T2/T3 | 41 (40.6) | 34 (37.4) | 7 (70.0) |  |
| Lymph node metastasis |  |  |  | 0.983 |
| Absent | 71 (70.3) | 64 (70.3) | 7 (70.0) |  |
| Present | 30 (29.7) | 27 (29.7) | 3 (30.0) |  |
| TIL |  |  |  | **0.020** |
| <5% | 87 (86.1) | 80 (87.9) | 7 (70.0) |  |
| 5-10% | 8 (7.9) | 5 (5.5) | 3 (30.0) |  |
| ≥10% | 6 (5.9) | 6 (6.6) | 0 (0.0) |  |
| ER |  |  |  | 0.567 |
| Negative | 6 (5.9) | 5 (5.5) | 1 (10.0) |  |
| Positive | 95 (94.1) | 86 (94.5) | 9 (90.0) |  |
| PR |  |  |  | **0.003** |
| Negative | 17 (16.8) | 12 (13.2) | 5 (50.0) |  |
| Positive | 84 (83.2) | 79 (86.8) | 5 (50.0) |  |
| HER-2 |  |  |  | **<0.001** |
| Negative | 94 (93.1) | 88 (96.7) | 6 (60.0) |  |
| Positive | 7 (6.9) | 3 (3.3) | 4 (40.0) |  |
| Ki-67 labelling index |  |  |  | **<0.001** |
| ≤14 | 83 (82.2) | 79 (86.8) | 4 (40.0) |  |
| >14 | 18 (17.8) | 12 (13.2) | 6 (60.0) |  |
| Molecular type |  |  |  | **<0.001** |
| Luminal A | 77 (76.2) | 74 (81.3) | 3 (30.0) |  |
| Luminal B | 19 (18.8) | 13 (14.3) | 6 (60.0) |  |
| HER-2 | 1 (1.0) | 0 (0.0) | 1 (10.0) |  |
| TNBC | 4 (4.0) | 4 (4.4) | 0 (0.0) |  |
