# Supplementary material for: Expression of PD-L1 in breast invasive lobular carcinoma
Source: PLoS One. 2024 Oct 10;19(10):e0309170. doi: 10.1371/journal.pone.0309170 (PMC11466385; doi:10.1371/journal.pone.0309170)
Supplement: S3 Table — (DOCX) [file pone.0309170.s003.docx]

| **Supplementary Table 3.** Impact of PD-L1 status on tumor recurrence and patient death in ILC | | | | | | |
| --- | --- | --- | --- | --- | --- | --- |
| Parameters | Tumor recurrence | | | Patient death | | |
|  | No | Yes | p-value | No | Yes | p-value |
| PD-L1 22C3 TC |  |  | n/a |  |  | n/a |
| <1% | 94 (100.0) | 7 (100.0) |  | 97 (100.0) | 4 (100.0) |  |
| ≥1% | 0 (0.0) | 0 (0.0) |  | 0 (0.0) | 0 (0.0) |  |
| PD-L1 22C3 IC |  |  | 0.652 |  |  | 0.281 |
| <1% | 74 (78.7) | 5 (71.4) |  | 75 (77.3) | 4 (100.0) |  |
| ≥1% | 20 (21.3) | 2 (28.6) |  | 22 (22.7) | 0 (0.0) |  |
| PD-L1 SP142 TC |  |  | n/a |  |  | n/a |
| <1% | 94 (100.0) | 7 (100.0) |  | 97 (100.0) | 4 (100.0) |  |
| ≥1% | 0 (0.0) | 0 (0.0) |  | 0 (0.0) | 0 (0.0) |  |
| PD-L1 SP142 IC |  |  | n/a |  |  | n/a |
| <1% | 94 (100.0) | 7 (100.0) |  | 97 (100.0) | 4 (100.0) |  |
| ≥1% | 0 (0.0) | 0 (0.0) |  | 0 (0.0) | 0 (0.0) |  |
| PD-L1 SP263 TC |  |  | 0.134 |  |  | 0.772 |
| <1% | 93 (98.9) | 6 (85.7) |  | 95 (97.9) | 4 (100.0) |  |
| ≥1% | 1 (1.1) | 1 (14.3) |  | 2 (2.1) | 0 (0.0) |  |
| PD-L1 SP263 IC |  |  | 0.454 |  |  | 0.578 |
| <1% | 87 (92.6) | 7 (100.0) |  | 90 (92.8) | 4 (100.0) |  |
| ≥1% | 7 (7.4) | 0 (0.0) |  | 7 (7.2) | 0 (0.0) |  |
